# Bevacizumab neutralizes the protective effect of vascular endothelial growth factor on retinal ganglion cells

**Published:** 2010-09-12

**Authors:** Vikram S. Brar, Rajesh K. Sharma, Ravi K. Murthy, K.V. Chalam

**Affiliations:** University of Florida College of Medicine, Department of Ophthalmology Jacksonville, FL

## Abstract

**Purpose:**

Vascular endothelial growth factor (VEGF) is well known for its role in pathologic neovascularization, including wet age-related macular degeneration. However, a growing body of evidence indicates that VEGF is also neuroprotective of non-vascular cells in various animal models through reduction of oxidative stress. In light of the widespread use of intraocular anti-VEGF therapies for age-related macular degeneration (AMD), we evaluated the impact of anti-VEGF agents on the neuroprotective effect of VEGF on retinal ganglion cells.

**Methods:**

Staurosporine differentiated retinal ganglion cells were treated with increasing doses of VEGF in the presence of hydrogen peroxide. After optimization, an increasing concentration of bevacizumab was added to neutralize VEGF-mediated protection. The degree of oxidative damage was measured at various time points using buthionine sulfoxime (BSO), a glutathione reductase inhibitor. Cell viability was assessed using WST-1 and Crystal violet assays.

**Results:**

VEGF (200 ng/ml) protected differentiated retinal ganglion cells (RGC)-5 against H_2_0_2_-mediated oxidative stress. This effect was eliminated by co-treatment with bevacizumab (2.0 mg/ml), which by itself was not cytotoxic.

**Conclusions:**

These results indicate an important role for VEGF in the maintenance of retinal ganglion cells.

## Introduction

Vascular endothelial growth factor (VEGF), a secreted 46 kDa glycoprotein, is an endothelial cell-specific angiogenic as well as a vasopermeable factor [1,2]. Although initially thought to be endothelial-specific, VEGF plays a major role during the development and maturation of neural tissue, including the retina [3]. During development, VEGF is expressed by astrocytes in the retinal ganglion cell layer, by cells of inner nuclear layer, Müller cells, and retinal pigment epithelial cells [4,5]. In the adult retina, VEGF is expressed in the absence of active neovascularization and is implicated in the maintenance and function of adult retina neuronal cells [6]. VEGF-A, an isoform of VEGF, reduces apoptosis in retinal neurons after ischemic injury and delays degeneration of retinal ganglion cells after axotomy [7]. Upregulation of VEGF occurs in many ischemic conditions of the retina, including age-related macular degeneration (AMD) and diabetic retinopathy [8,9]. Higher intraocular VEGF levels lead to subretinal and vitreous hemorrhage, retinal detachment, and often blindness [10].

Intravitreal injections of anti-VEGF agents such as bevacizumab are widely used in the treatment of AMD to reduce the angiogenesis associated with choroidal neovascularization [11,12]. Hypothetically, repeated treatment with anti-VEGF agents should interfere with the neuroprotective action of VEGF. Recently, Saint-Geniez et al. [6] have shown in animal studies that small, interfering RNA (siRNA)-mediated gene silencing of VEGF led to a reduction in the thickness of retinal cell layers. They concluded that VEGF has a neuroprotective role in the survival of retinal neurons. In this study, we tested this hypothesis in a cell culture model by investigating the neuroprotective effect of VEGF on differentiated rat retinal ganglion cell, RGC-5 after oxidative stress, and whether treatment with bevacizumab can abrogate this effect. We used the oxidative stress model to replicate the in vivo conditions, which are implicated in the pathogenesis of macular degeneration. Further, we found that retinal ischemic and oxidative injury leads to increased levels of VEGF, resulting in angiogenesis.

## Methods

### Cell culture

Rat retinal ganglion cells (RGC-5; Molecular Brain Research 86 [2001]) [1–12] were supplied courtesy of Dr. Neeraj Agarwal (UNT Health Science Center, Fort Worth, TX) and were cultured in Dubelco’s Modified Eagle Medium (DMEM: Invitrogen, Carlsbad, CA), containing 10% fetal bovine serum (FBS) and 100 U/ml penicillin/100 μg/ml streptomycin. All cells were maintained in logarithmic growth in T-75 flaskware and incubated at 37 °C in a 95% air and 5% CO_2_ environment.

### Differentiation

RGC-5 cells were differentiated by treating the cells with Staurosporine using a technique previously described [13]. Morphologic evidence of a neuron cell line was determined using phase contrast bright-field microscopy. Further, immunocytochemistry was performed using a monoclonal antibody against class III β-tubulin to confirm differential expression of markers of neuronal differentiation.

### Oxidative stress

Different concentrations of hydrogen peroxide (H_2_O_2_) were generated using serial dilution and applied to the Staurosporine-differentiated RGCs for 24 h to generate a dose response curve. Cell numbers were assessed using a Water Soluble Tetrazolium (WST)-1 (Roche, Mannheim, Germany) assay. Briefly, WST-1 is a tetrazolium salt that is cleaved by mitochondrial dehydrogenases in viable cells, producing a proportionate color change. Plates were then read on a spectrophotometer at 440 nm with reference wavelength at 690 nm.

Subsequently, differentiated cells were treated with increasing concentrations of human VEGF (hVEGF165; Invitrogen) in adjacent wells with alternate wells containing 800 µM H_2_O_2_ for 24 h. The difference in cell number between H_2_O_2_-treated and untreated wells were compared, using the WST-1 assay as described above. The aforementioned experiment was repeated in the presence of 25 μM buthionine sulfoxime (BSO), an inhibitor of glutathione (GSH) synthesis, thereby enhancing oxidative stress in our model. Cells were exposed to 25 μM BSO for 24 h following attachment and differentiation. Increasing concentrations of hVEGF165 were applied, followed by 24 h exposure to 800 μM H_2_O_2_.

To evaluate the effect of bevacizumab blockade of hVEGF165, we first conducted cytotoxicity experiments to explore any potential effect in our differentiated model. Cells were plated in 96-well plates at 2,000 cells/well, allowed to attach for 24 h, and subsequently differentiated with Staurosporine. Serial dilutions of commercially available bevacizumab (Avastin®: Genentech, San Francisco, CA) were performed to obtain treatment concentrations of 0.1, 1.0, and 2.0 mg/ml, and were applied for 24 h. H_2_O_2_ (1 mM) served as a positive control. Cell numbers were assessed using WST-1. Parallel experiments were conducted in a similar fashion and evaluated with crystal violet staining (CV). In brief, cells were fixed for 10 min in 4% formaldehyde, rinsed twice with PBS and dried. Cells were stained with 0.1% crystal violet solution for 30 min. Excess stain was washed once with PBS and twice with distilled water. Plates were dried again and then bound. Stain was dissolved in 100 μl of 10% acetic acid solution per well. Plates were read in a plate-reader with a 560 nm filter.

In subsequent experiments, cells were plated in 96-well plates and differentiated as described above. Increasing concentrations of bevacizumab were added to wells containing 200 ng VEGF165 and wells containing VEGF and 800 µM H_2_O_2_. Cell numbers using the WST-1 assay were determined following 24 h exposure.

## Results

### Differentiation

**Figure 1:**
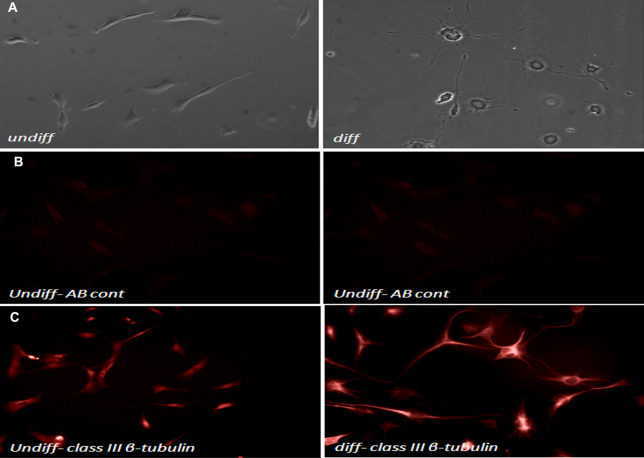
Morphological and immunohistochemical differentiation of retinal ganglion cell (RGC)-5 cells. **A**: Phase contrast biomicroscopy comparing undifferentiated with differentiated RGC-5 following exposure to Staurosporine. Note the elongated neuronal processes in the treated cells. **B**: Antibody control for immunocytochemistry staining **C**: Immunocytochemistry staining for class III β-tubulin highlights the elongated processes in Staurosporine treated cells. undiff: undifferentiated, diff: differentiated, AB: antibody control.

Representative bright-field microscopy images demonstrate morphologic changes between differentiated and undifferentiated RGC-5 cells (Figure 1A).

Immunocytochemistry demonstrates strong staining for class III β-tubulin, which highlights the elongated neuronal processes (Figure 1B, Antibody control and Figure 1C).

### Oxidative stress

**Figure 2:**
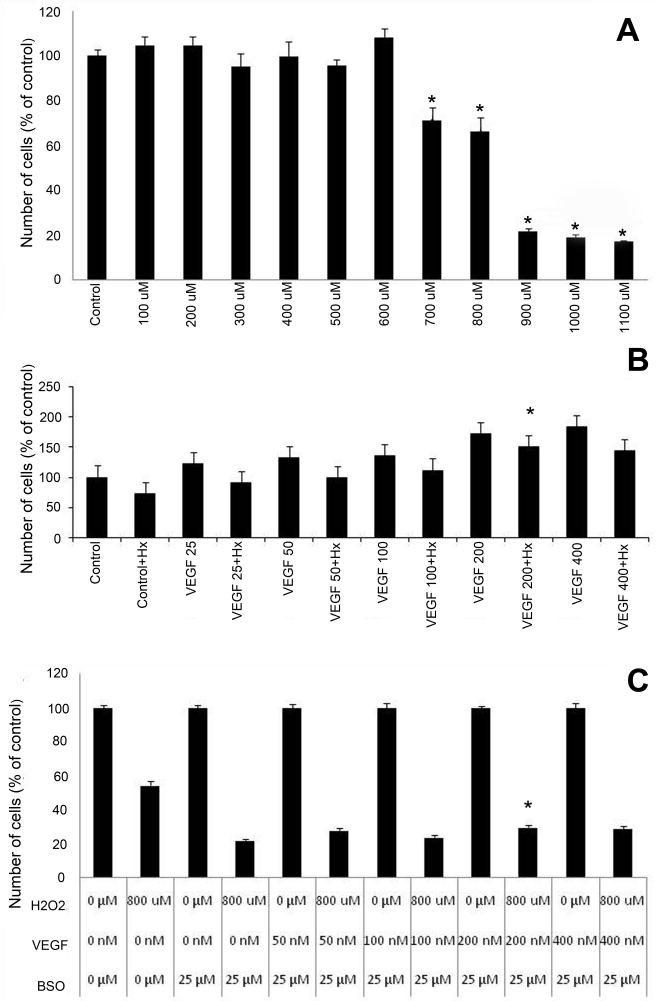
Effects of vascular endothelial growth factor (VEGF) in oxidative stress induced cytotoxicity in retinal ganglion cell (RGC)-5 cells. **A**: Hydrogen p 258 eroxide dose response curve, following 24 h exposure, revealed 30%–35% reduction in cell numbers at 700 and 800 μM doses. Higher doses resulted in 80% reduction (*p-value<0.005). **B**: VEGF-mediated protection was evaluated in parallel wells, where hydrogen peroxide (800 μM)-treated cells are compared with cells treated with VEGF alone. There was a reduction in percentage decrease of cell numbers when cells were co-treated with VEGF and H_2_O_2_, compared with controls administered in a dose-dependent manner with increasing doses of VEGF. The maximum protection was provided at the 200 nM dose (p=0.001). **C**: Treatment with the glutathione reductase inhibitor buthionine sulfoxime (BSO) increased H_2_O_2_-mediated reduction in cell numbers by 22%. hVEGF165 at 200 ng/ml provided the greatest protection, increasing cell numbers by 8%, p<0.0005. Cell numbers were determined using a Water Soluble Tetrazolium (WST)-1 assay and are expressed as percent of control. H_2_O_2_: hydrogen peroxide.

Twenty-four h H_2_O_2_ dose response studies identified 800 μM as the ideal concentration, reliably producing a 40% reduction in cell numbers compared with control (Figure 2A). This dose was used in subsequent experiments to test VEGF protection. 200 ng/ml of VEGF165 afforded the greatest protection to oxidative stress induced by 24 h exposure to 800 μM H_2_O_2_. There was a reduction in percentage decrease of cell numbers when cells were co-treated with VEGF and H_2_O_2_, compared with controls administered in a dose-dependent manner with increasing doses of VEGF. The maximum protection was provided at the 200 nM dose (p=0.001; Figure 2B). BSO increased H_2_O_2_-mediated reduction in cell numbers by 22%. hVEGF165 at 200 ng/mL provided the greatest protection, increasing cell numbers by 8%, p<0.0005 (Figure 2C).

**Figure 3:**
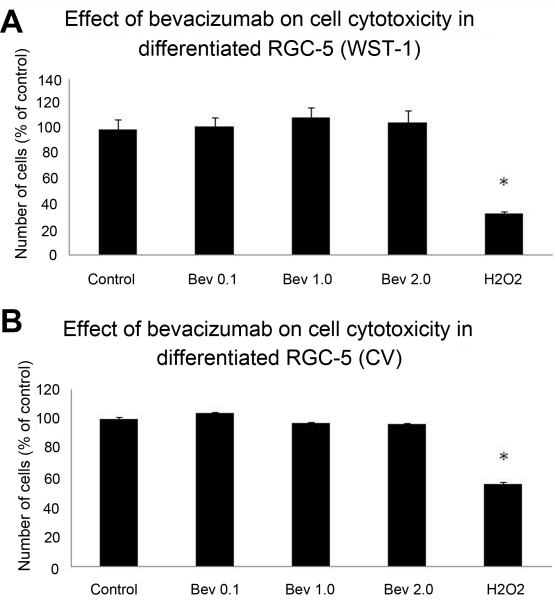
Effect of bevacizumab, following 24 h exposure, did not reveal evidence of cytotoxicity at any of the doses tested. Hydrogen peroxide (1 mM) served as a positive control. Cell numbers were assessed using Water Soluble Tetrazolium (WST)-1 (**A**) and crystal violet assays (**B**), and are expressed as percent of the control (*p- value<0.001). Bev: Bevacizumab. Error bars are SEM n=8 for all experiments.

**Figure 4:**
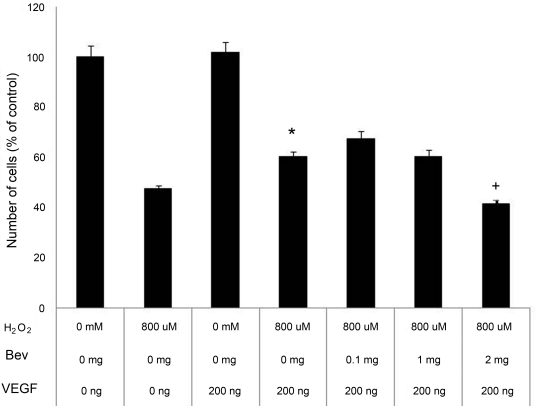
Effect of bevacizumab on retinal ganglion cell (RGC)-5 viability. Vascular endothelial growth factor (VEGF) protected against hydrogen peroxide-mediated oxidative stress compared with untreated controls, *p-value <0.005. This effect was eliminated by using bevacizumab at the 2 mg/ml dose, p-value <0.001. Cell numbers were determined using the Water Soluble Tetrazolium (WST)-1 assay and are expressed as percent of control. H_2_O_2_: hydrogen peroxide, Bev: bevacizumab. Error bars are SEM n=8 for all experiments.

Bevacizumab was not cytotoxic at any of the doses tested (p-values 0.34–0.69). This result was reflected in both WST-1 and CV assays (Figure 3A,B, respectively). H_2_O_2_ served as a positive control and consistently reduced cell numbers by 50%, p<0.0005. hVEGF165 afforded a 12% increase in cell numbers compared with control cells exposed to 800 μM H_2_O_2_, p<0.005. This effect was completely blocked by bevacizumab at 2.0 mg/ml, returning cell numbers to near control levels (Figure 4).

## Discussion

Anti-VEGF therapy plays a significant role in the management of retinal and retinal vascular disorders [9–12]. Target therapy, though aimed against the angiogenic effect of VEGF, can reduce the potential benefit of VEGF in neuroprotection from oxidative stress. In our experiments, we provide evidence to suggest that addition of the VEGF inhibitor bevacizumab, while not toxic to differentiated RGC-5 at clinically relevant doses, blocked the protective effect of VEGF in our in vitro model.

In the human eye, there is increasing evidence that pathogenic oxidative mechanisms contribute to the progression of pathological conditions such as age-related macular degeneration and glaucoma [14–18]. Phagocytosis of outer segments by the retinal pigment epithelium results in the generation of reactive oxygen species such as superoxide anion, hydroxyl, and hydrogen peroxide [19]. In different cell types, including vascular smooth muscles, VEGF expression is triggered under the influence of oxidative stress. Castilla et al. have demonstrated an increased VEGF expression in aortic endothelial cells that were exposed to cytotoxic agents such as hydrogen peroxide, cytochalasin, and cyclosporine [20]. In this study, we used H_2_O_2_ to induce oxidative stress to replicate the in vivo conditions that are implicated in the pathogenesis of AMD.

The development of the transformed rat RGC-5 provided a constant supply of cells for laboratory investigation, although their lack of morphologic characteristics of neurons has raised debate [13]. A recent report describes the process of differentiation of this cell line with the receptor tyrosine kinase inhibitor Staurosporine. Treatment with Staurosporine resulted in elongated neuronal processes imitating those present in vivo, compared with the control RGC-5 [13]. Our experiments further characterized this model, demonstrating differential expression of class III β-tublin, which highlighted the morphologic changes. This result thus provided an in vitro model that better approximates retinal ganglion cells in vivo.

Several mechanisms have been elaborated to explain the cytoprotective effect mediated by VEGF against oxidative stress [21,22]. In a study evaluating the role of growth factor-modulated antioxidant expression, Madhavan et al. [21] found elevated expression of the antioxidant enzyme superoxide dismutase 2 (SOD2). This enzyme protected neuronal cells from oxidative stress when exposed to 3-NP, an inhibitor of mitochondrial respiratory complex, in the presence of VEGF. Similarly, in animal models of hyperoxic acute lung injury, VEGF has been shown to protect vascular endothelial cells through induction of heme oxygenase-1 (HO-1) [22]. In this study, we demonstrated protection of differentiated retinal ganglion cells by hVEGF165 from oxidative stress induced by H2O2. Experiments using the glutathione reductase inhibitor BSO blunted this effect, indicating a potential role of glutathione in VEGF-mediated cytoprotection. An alternative explanation is that the oxidative stress was more robust in cells where endogenous reducing agents have been depleted, and any minimal cytoprotective action of VEGF was no longer evident.

Our conclusions are drawn from in vitro studies, which are at best approximate and cannot mimic in vivo conditions. Further, only one VEGF isoform was evaluated. Treatment with different isoforms may have highlighted differences between specific and non-specific VEGF inhibition. Lastly, the increased presence of class III β-tubulin provides evidence of neuronal elements, but this is not specific for retinal ganglion cells.

In summary, we offer in vitro evidence that VEGF protection against oxidative stress is blocked by bevacizumab, in a model of differentiated retinal ganglion cells.
